# Prevalence of Alcohol Dependence Among US Adult Drinkers, 2009–2011

**DOI:** 10.5888/pcd11.140329

**Published:** 2014-11-20

**Authors:** Marissa B. Esser, Sarra L. Hedden, Dafna Kanny, Robert D. Brewer, Joseph C. Gfroerer, Timothy S. Naimi

**Affiliations:** Author Affiliations: Marissa B. Esser, Robert D. Brewer, Centers for Disease Control and Prevention, Atlanta, Georgia; Sarra L. Hedden, Joseph C. Gfroerer, Center for Behavior Health Statistics and Quality, Substance Abuse and Mental Health Services Administration, Rockville, Maryland; Timothy S. Naimi, Boston University Medical Center, Boston, Massachusetts.

## Abstract

**Introduction:**

Excessive alcohol consumption is responsible for 88,000 deaths annually and cost the United States $223.5 billion in 2006. It is often assumed that most excessive drinkers are alcohol dependent. However, few studies have examined the prevalence of alcohol dependence among excessive drinkers. The objective of this study was to update prior estimates of the prevalence of alcohol dependence among US adult drinkers.

**Methods:**

Data were analyzed from the 138,100 adults who responded to the National Survey on Drug Use and Health in 2009, 2010, or 2011. Drinking patterns (ie, past-year drinking, excessive drinking, and binge drinking) were assessed by sociodemographic characteristics and alcohol dependence (assessed through self-reported survey responses and defined as meeting ≥3 of 7 criteria for dependence in the Diagnostic and Statistical Manual of Mental Disorders, Fourth Edition).

**Results:**

Excessive drinking, binge drinking, and alcohol dependence were most common among men and those aged 18 to 24. Binge drinking was most common among those with annual family incomes of $75,000 or more, whereas alcohol dependence was most common among those with annual family incomes of less than $25,000. The prevalence of alcohol dependence was 10.2% among excessive drinkers, 10.5% among binge drinkers, and 1.3% among non-binge drinkers. A positive relationship was found between alcohol dependence and binge drinking frequency.

**Conclusion:**

Most excessive drinkers (90%) did not meet the criteria for alcohol dependence. A comprehensive approach to reducing excessive drinking that emphasizes evidence-based policy strategies and clinical preventive services could have an impact on reducing excessive drinking in addition to focusing on the implementation of addiction treatment services.

## MEDSCAPE CME

Medscape, LLC is pleased to provide online continuing medical education (CME) for this journal article, allowing clinicians the opportunity to earn CME credit.

This activity has been planned and implemented in accordance with the Essential Areas and policies of the Accreditation Council for Continuing Medical Education through the joint sponsorship of Medscape, LLC and Preventing Chronic Disease. Medscape, LLC is accredited by the ACCME to provide continuing medical education for physicians.

Medscape, LLC designates this Journal-based CME activity for a maximum of 1 **AMA PRA Category 1 Credit(s)™**. Physicians should claim only the credit commensurate with the extent of their participation in the activity.

All other clinicians completing this activity will be issued a certificate of participation. To participate in this journal CME activity: (1) review the learning objectives and author disclosures; (2) study the education content; (3) take the post-test with a 75% minimum passing score and complete the evaluation at www.medscape.org/journal/pcd (4) view/print certificate.

**Release date: November 20, 2014; Expiration date: November 20, 2015**

### Learning Objectives

Upon completion of this activity, participants will be able to:

- Describe excessive drinking and clinical correlates among adult drinkers in the United States, based on a database study of survey responses
- Describe binge drinking and clinical correlates among adult drinkers in the United States
- Describe alcohol dependence and clinical correlates among adult drinkers in the United States

**EDITORS**

Ellen Taratus, Editor, *Preventing Chronic Disease*. Disclosure: Ellen Taratus has disclosed no relevant financial relationships.

**CME AUTHOR**

Laurie Barclay, MD, Freelance writer and reviewer, Medscape, LLC. Disclosure: Laurie Barclay, MD, has disclosed no relevant financial relationships.

**AUTHORS AND CREDENTIALS**

Disclosures: Marissa Esser, MPH; Sarra L. Hedden, PhD; Dafna Kanny, PhD; Robert Brewer, MD, MSPH; Joseph Gfroerer, BA; and Timothy S. Naimi, MD, MPH have disclosed no relevant financial relationships.

Affiliations: Dafna Kanny, Marissa Esser, Robert Brewer, Centers for Disease Control and Prevention, Atlanta, Georgia; Sarra L. Hedden, Joseph Gfroerer, Center for Behavior Health Statistics and Quality, Substance Abuse and Mental Health Services Administration, Rockville, Maryland; Timothy S. Naimi, Boston University Medical Center, Boston, Massachusetts.

## Introduction

Excessive alcohol consumption is responsible for an average of 88,000 deaths each year and cost the United States $223.5 billion in 2006 (1,2). Half of these deaths and three-quarters of the economic costs are due to binge drinking (ie, ≥4 drinks for women and ≥5 drinks for men in a single occasion) (1,3). Binge drinking is also associated with a myriad of health and social problems (eg, violence, new HIV infections, unintended pregnancies, and alcohol dependence) (4,5).

It is often assumed that most excessive drinkers are alcohol dependent. However, few studies have examined the prevalence of alcohol dependence among excessive drinkers — specifically among binge drinkers. Access to such information is important to inform the prioritization of strategies to prevent excessive drinking and treat alcohol dependence. Because binge drinkers are at higher risk than non-binge drinkers for alcohol dependence (6), population-based policies offer an important way to prevent alcohol dependence. The clinical diagnosis of alcohol dependence is based on criteria in the Diagnostic and Statistical Manual of Mental Disorders (DSM), Fourth Edition, and involves the assessment of warning signs such as tolerance, withdrawal, impaired control, and unsuccessful attempts to cut down (7).

Data from the 2002 New Mexico Behavioral Risk Factor Surveillance System (BRFSS) found that 10.7% of excessive drinkers (ie, binge drinkers, heavy drinkers, or alcohol-impaired drivers) and 8.1% of binge drinkers met diagnostic criteria for alcohol dependence (8). A study of the 2001–2002 National Epidemiologic Survey of Alcohol-Related Conditions (NESARC) found that the prevalence of alcohol dependence among binge drinkers aged 18 or older was 4.4% among those with 1 to 11 binge drinking occasions in the previous year and jumped to 21.9% among those with 12 or more binge drinking occasions in the previous year (9).

There are no current estimates of the prevalence of alcohol dependence among US adults based on patterns of drinking. The objective of this study was to update prior estimates of the prevalence of alcohol dependence among US adult drinkers. If most binge drinkers do not meet the diagnostic criteria for alcohol dependence, evidence-based policy strategies and clinical preventive services may effectively reduce binge drinking in most cases without requiring addiction treatment.

## Methods

### Sample and procedures

The National Survey on Drug Use and Health (NSDUH) is a series of cross-sectional surveys of a nationally representative sample of noninstitutionalized US residents aged 12 years or older in the 50 states and Washington, DC. Conducted by the Substance Abuse and Mental Health Services Administration, the NSDUH is designed to assess the prevalence and correlates of alcohol and other drug use disorders in the general US population. A multistage probability sample was independently selected for each state and Washington, DC. Interviews were conducted using a computer-assisted personal interview and audio computer-assisted self-interviewing to enhance respondent’s privacy during visits to households and group living facilities. Respondents were compensated $30 for their participation in the survey.

Data for this study were pooled from surveys in 2009, 2010 (revised in March 2012), and 2011, and a restricted-use data set was created for this collaborative study between the Centers for Disease Control and Prevention and the Substance Abuse and Mental Health Services Administration. A new population weight was created for this multiyear data set by dividing the original weights for the single-year data sets by 3. This new population weight was then used to produce annual average estimates of the US adult civilian noninstitutionalized population aged 18 or older. Weighted response rates for the computer-assisted interviews were 75.7% in 2009, 74.7% in 2010, and 74.4% in 2011. The final combined sample consisted of approximately 138,100 adult respondents from all 50 states and Washington, DC. Further description of the methods for the NSDUH has been published (10). This study did not involve human research participants and was exempt from institutional review board oversight.

### Measures

Past-year drinking was defined as consuming at least 1 drink in the past 12 months. Excessive drinking was defined as binge drinking; heavy drinking; any past 30-day drinking by respondents aged 18 to 20 (who are under the minimum legal drinking age) if not already included in another drinking category; or any past 30-day alcohol consumption by pregnant women (1). The NSDUH consists of core and noncore questions. The core questions do not change from year to year to preserve the ability to assess trends over time. The noncore modules are less static and are asked in the survey after the core-modules. Only respondents who reported ever having an alcoholic beverage were asked the noncore alcohol consumption questions. Binge drinking for men was defined as a nonzero response to the following question in the core portion of the survey: “During the past 30 days, that is, since [DATEFILL], on how many days did you have 5 or more drinks on the same occasion?” Binge drinking for women was defined as a nonzero response to the following question in the noncore portion of the survey: “During the past 30 days, that is, since [DATEFILL], on how many days did you have 4 or more drinks on the same occasion?” Heavy drinking was defined as 8 or more drinks per week during the past 30 days for women or 15 or more drinks per week for men (1).

Alcohol dependence was defined as past-year drinking, 3 or more (of 7) dependence criteria, and consuming at least 1 drink on 6 or more days in the past 12 months (11). The alcohol dependence questions in the NSDUH align with the diagnostic criteria for alcohol dependence in the fourth edition of the DSM (DSM-IV) (7). These include tolerance, withdrawal, impaired control, unsuccessful attempts to cut down or stop drinking, continued use despite problems, neglect of activities, and time spent in alcohol-related activity. The classification of alcohol dependence in this study is based on self-reported responses to the NSDUH and is not based on a diagnosis in a clinical setting or from medical records; therefore, alcohol dependence in this study is based on respondents’ survey data.

Sociodemographic characteristics assessed in this study were sex, age group (18–24, 25–34, 35–44, 45–64, ≥65), race/ethnicity (non-Hispanic white, non-Hispanic black, American Indian or Alaskan Native, Native Hawaiian or other Pacific Islander, Asian, ≥2 races/ethnicities, Hispanic or Latino), education level attained (<high school, high school, some college, college graduate), annual family income (<$25,000, $25,000 to <$50,000, $50,000 to <$75,000, ≥$75,000), and employment status (full-time, part-time, unemployed, other).

### Statistical analysis

We conducted analyses using SAS-callable SUDAAN version 10 (SAS, SAS Institute Inc; SUDAAN, RTI International). NSDUH weights and survey design variables were used in the computation of all prevalence estimates and 95% confidence intervals (CIs). As part of NSDUH’s editing and imputation process, inconsistent or missing data for the core variables and many sociodemographic variables were statistically imputed using predictive mean neighborhood imputation (12). Respondents with missing data on variables that were not imputed were excluded from the analysis; however, item nonresponse for each of these variables was less than 2%. Estimated weighted numbers are reported in thousands, and sample sizes are rounded to the nearest hundred.

## Results

From 2009 to 2011, the prevalence of past-year drinking among adults was 70.5% (95% CI, 70.0%–70.9%), the prevalence of past-month excessive drinking was 29.3% (95% CI, 28.9%–29.7%), the prevalence of past-month binge drinking was 27.4% (95% CI, 27.0%–27.8%), and the prevalence of DSM-IV alcohol dependence among all respondents was 3.5% (95% CI, 3.3%–3.6%) (Table 1). The prevalence of excessive drinking, binge drinking, and alcohol dependence were highest among men, those aged 18 to 24, and those who were unemployed. Binge drinking was more common among Native Hawaiians or other Pacific Islanders (31.8%; 95% CI, 24.1%–40.6%) and non-Hispanic whites (28.6%; 95% CI, 28.1%–29.1%) than among other racial/ethnic groups, but most of these differences were not significant. In contrast, the prevalence of alcohol dependence among current drinkers was significantly higher among American Indians or Alaskan Natives (9.0%; 95% CI, 6.8%–11.8%) relative to other racial/ethnic groups.

**Table 1:** Prevalence of Drinking Patterns Among US Adults,^a^ by Sociodemographic Characteristics, National Survey on Drug Use and Health, 2009–2011

Sociodemographic Characteristic	Past-Year Drinking^b^		Past-Month Excessive Drinking^c^		Past-Month Binge Drinking^d^		Past-Year Alcohol Dependence^e^	
	n^f^	% (95% CI)	n^f^	% (95% CI)	n^f^	% (95% CI)	n^f^	% (95% CI)
**Overall**	138,000	70.5 (70.0–70.9)	136,900	29.3 (28.9–29.7)	138,100	27.4 (27.0–27.8)	137,400	3.5 (3.3–3.6)
**Sex**								
Men	65,000	74.6 (73.9–75.2)	64,500	35.3 (34.7–35.9)	65,000	33.8 (33.2–34.5)	64,600	4.5 (4.2–4.7)
Women	73,100	66.6 (66.0–67.3)	72,400	23.7 (23.2–24.2)	73,100	21.4 (21.0–21.9)	72,700	2.5 (2.4–2.7)
**Age group, y**								
18–24	60,400	77.5 (76.9–78.0)	59,900	49.6 (48.9–50.3)	60,400	43.4 (42.7–44.1)	59,900	6.5 (6.2–6.8)
25–34	27,900	79.8 (79.1–80.5)	27,700	41.0 (40.1–41.9)	27,900	40.2 (39.3–41.1)	27,800	5.4 (5.0–5.8)
35–44	18,400	76.2 (75.4–77.0)	18,300	31.3 (30.4–32.1)	18,400	30.4 (29.6–31.3)	18,400	3.7 (3.4–4.1)
45–64	23,200	69.6 (68.7–70.5)	23,000	23.4 (22.7–24.2)	23,200	22.1 (21.4–22.8)	23,100	2.6 (2.3–2.9)
≥65	8,100	51.1 (49.6–52.7)	8,000	11.5 (10.6–12.4)	8,100	9.7 (8.8–10.5)	8,100	0.7 (0.5–1.0)
**Race/ethnicity**								
Non-Hispanic white	89,900	74.6 (74.0–75.1)	89,300	30.6 (30.1–31.1)	89,900	28.6 (28.1–29.1)	89,600	3.4 (3.2–3.6)
Non-Hispanic black	16,400	62.0 (60.6–63.4)	16,200	25.3 (24.2–26.5)	16,400	23.6 (22.5–24.7)	16,300	3.7 (3.3–4.1)
AI/AN	1,900	57.3 (51.7–62.7)	1,900	27.7 (23.8–31.8)	1,900	26.7 (23.0–30.7)	1,900	9.0 (6.8–11.8)
NH/PI	600	65.6 (57.1–73.2)	500	34.0 (26.1–42.8)	600	31.8 (24.1–40.6)	500	3.5 (1.8–6.8)
Asian	4,900	55.5 (52.8–58.2)	4,900	15.7 (14.2–17.3)	4,900	14.2 (12.8–15.7)	4,900	1.4 (1.1–1.8)
≥2 Races/ethnicities	3,600	68.9 (65.1–72.4)	3,600	28.3 (25.6–31.3)	3,600	26.0 (23.4–28.8)	3,600	4.1 (3.1–5.3)
Hispanic or Latino	20,700	63.2 (61.9–64.5)	20,500	30.9 (29.8–32.0)	20,700	29.4 (28.3–30.5)	20,500	4.0 (3.6–4.4)
**Education**								
<High school	21,700	50.6 (49.4–51.8)	21,500	26.0 (25.1–26.9)	21,700	24.5 (23.6–25.4)	21,500	4.3 (3.9–4.8)
High school	44,900	65.9 (65.1–66.7)	44,500	30.0 (29.3–30.7)	44,900	28.0 (27.3–28.7)	44,600	3.3 (3.1–3.6)
Some college	40,700	75.7 (74.9–76.5)	40,400	32.3 (31.5–33.0)	40,700	30.1 (29.3–30.8)	40,600	4.0 (3.8–4.4)
College graduate	30,800	80.7 (80.0–81.5)	30,600	27.6 (26.8–28.4)	30,800	26.0 (25.2–26.7)	30,700	2.6 (2.4–2.9)
**Annual family income, $**								
<25,000	43,600	57.7 (56.7–58.6)	43,200	28.5 (27.6–29.3)	43,600	26.7 (25.9–27.5)	43,300	4.8 (4.5–5.2)
25,000 to <50,000	38,300	66.3 (65.5–67.2)	37,900	28.4 (27.6–29.2)	38,300	26.7 (26.0–27.5)	38,100	3.3 (3.1–3.6)
50,000 to <75,000	21,900	73.8 (72.8–74.9)	21,700	28.7 (27.8–29.6)	21,900	27.0 (26.1–27.9)	21,800	2.9 (2.6–3.2)
≥75,000	34,300	82.1 (81.3–82.8)	34,000	31.1 (30.3–31.9)	34,300	28.8 (28.1–29.6)	34,200	2.8 (2.6–3.1)
**Employment status**								
Full-time	66,000	78.4 (77.8–78.9)	65,600	33.8 (33.3–34.4)	66,000	32.6 (32.0–33.1)	65,800	3.5 (3.3–3.7)
Part-time	27,100	74.1 (73.0–75.2)	26,900	32.1 (31.1–33.1)	27,100	28.8 (27.8–29.7)	27,000	4.0 (3.7–4.4)
Unemployed	12,800	72.3 (70.7–73.7)	12,600	38.1 (36.7–39.6)	12,800	35.5 (34.0–36.9)	12,700	6.3 (5.6–7.0)
Other^g^	32,100	54.9 (53.9–55.9)	31,800	18.4 (17.7–19.1)	32,100	16.4 (15.7–17.0)	32,000	2.5 (2.3–2.8)

Abbreviations: CI, confidence interval; AI/AN, American Indian or Alaskan Native; NH/PI, Native Hawaiian or other Pacific Islander.

a All respondents to National Survey on Drug Use and Health, 2009–2011 (data set used for 2010 was revised in March 2012).

b Adults who reported drinking at least 1 drink in the past 12 months.

c Adults who reported drinking 15 or more drinks per week (men) or 8 or more drinks per week (women) in the past 30 days; reported drinking 5 or more drinks (men) or 4 or more drinks (women) during an occasion on at least 1 day in the past 30 days; reported any drinking (adults aged 18–20) in the past 30 days and not included in the category of binge drinking; or reported any alcohol consumption and pregnancy (women) in the past 30 days.

d Adults who reported consuming 5 or more drinks (men) or 4 or more drinks (women), per occasion, on at least 1 day in the past 30 days.

e Adults who met at least 3 of 7 of the DSM-IV (Diagnostic and Statistical Manual of Mental Disorders, Fourth Edition) criteria for alcohol dependence (ie, tolerance, withdrawal, impaired control, unsuccessful attempts to cut down or stop drinking, continued use despite problems, neglect of activities, time spent in alcohol-related activity) and consumed 1 or more drinks on 6 or more days in the past 12 months. Respondents whose information was unknown were excluded.

f Sample sizes of the number of survey respondents (rounded to the nearest hundred).

g Students, people keeping house or caring for children full time, people who are retired or who have disabilities, or other people not in the labor force.

When evaluated by education, the prevalence of binge drinking was significantly higher among those with some college education (30.1%; 95% CI, 29.3%–30.8%) than among those with other levels of education. However, the prevalence of alcohol dependence among those with less than a high school education (4.3%; 95% CI, 3.9%–4.8%) was significantly higher than among those with a high school education or college education but not higher than the prevalence among those with some college. The prevalence of binge drinking was also significantly higher among those with an annual family income of $75,000 or more (28.8%; 95% CI, 28.1%–29.6%) than among those with lower family incomes, whereas the prevalence of alcohol dependence was significantly higher among those with an annual family income of less than $25,000 (4.8%; 95% CI, 4.5%–5.2%) than among those in other income groups (Table 1).

When evaluated by drinking pattern, the prevalence of alcohol dependence was 10.2% (95% CI, 9.8%–10.6%) among excessive drinkers, 10.5% (95% CI, 10.1%–11.0%) among binge drinkers, and 1.3% (95% CI, 1.2%–1.5%) among non-binge drinkers (Table 2). The prevalence of alcohol dependence was significantly higher among excessive drinkers and binge drinkers than among non-binge drinkers across all sociodemographic groups. In contrast, the prevalence of alcohol dependence was similar among excessive drinkers and binge drinkers across most groups except those aged 18 to 24, among whom the prevalence of alcohol dependence was significantly higher among binge drinkers (13.2%; 95% CI, 12.7%–13.8%) than among excessive drinkers (11.9%; 95% CI, 11.4%–12.4%).

**Table 2:** Prevalence of Alcohol Dependence^a^ Among US Adult Drinkers, by Drinking Pattern and Sociodemographic Characteristics, National Survey on Drug Use and Health, 2009–2011^b^

Sociodemographic Characteristic	Past-Month Excessive Drinkers^c^		Past-Month Binge Drinkers^d^		Past-Month Non-Binge Drinkers^e^	
	n^f^	% (95% CI)	n^f^	% (95% CI)	n^f^	% (95% CI)
**Overall**	54,100	10.2 (9.8–10.6)	50,200	10.5 (10.1–11.0)	53,400	1.3 (1.2–1.5)
**Sex**						
Men	29,400	10.9 (10.3–11.5)	27,700	11.1 (10.6–11.7)	22,800	1.7 (1.5–2.0)
Women	24,700	9.2 (8.6–9.8)	22,500	9.7 (9.0–10.3)	30,500	1.0 (0.8–1.2)
**Age group, y**						
18–24	29,900	11.9 (11.4–12.4)	26,500	13.2 (12.7–13.8)	20,200	2.3 (2.0–2.6)
25–34	11,800	11.2 (10.4–12.1)	11,700	11.3 (10.5–12.2)	10,700	2.1 (1.7–2.6)
35–44	5,800	10.0 (9.0–11.0)	5,700	10.0 (9.0–11.0)	8,300	1.5 (1.2–1.9)
45–64	5,800	9.1 (8.1–10.2)	5,600	9.4 (8.4–10.5)	10,800	1.1 (0.8–1.3)
≥65	900	5.6 (4.0–7.7)	800	5.3 (3.7–7.6)	3,300	0.5 (0.3–1.1)
**Race/ethnicity**						
Non-Hispanic white	37,700	9.8 (9.3–10.3)	35,200	10.1 (9.6–10.7)	36,000	1.1 (1.0–1.3)
Non-Hispanic black	5,100	12.6 (11.2–14.2)	4,600	13.1 (11.6–14.8)	6,400	1.6 (1.2–2.1)
AI/AN	700	27.5 (20.9–35.3)	700	28.1 (21.4–36.0)	600	5.0 (2.8–8.7)
NH/PI	200	4.4 (2.7–7.2)	200	4.5 (2.7–7.4)	200	—g
Asian	1,200	7.1 (5.4–9.2)	1,100	7.5 (5.7–9.8)	2,000	0.8 (0.4–1.4)
≥2 Races/ethnicities	1,600	11.4 (8.7–14.8)	1,400	11.8 (8.9–15.4)	1,400	2.3 (1.2–4.5)
Hispanic or Latino	7,600	10.4 (9.3–11.5)	7,000	10.6 (9.5–11.9)	6,800	2.5 (1.9–3.4)
**Education**						
<High school	7,500	13.7 (12.4–15.0)	6,900	14.2 (12.9–15.7)	6,200	3.2 (2.5–4.2)
High school	17,600	9.7 (9.0–10.5)	16,100	10.0 (9.2–10.8)	15,800	1.5 (1.2–1.7)
Some college	17,800	10.9 (10.1–11.7)	16,300	11.4 (10.6–12.3)	16,400	1.4 (1.1–1.7)
College graduate	11,200	8.3 (7.5–9.1)	11,000	8.5 (7.7–9.3)	14,900	0.8 (0.6–1.0)
**Annual family income, $**						
<25,000	17,800	14.2 (13.3–15.2)	16,300	14.9 (13.9–15.9)	13,900	2.8 (2.3–3.4)
25,000 to <50,000	14,500	10.1 (9.3–10.9)	13,600	10.4 (9.6–11.2)	14,300	1.4 (1.1–1.7)
50,000 to <75,000	8,200	9.0 (8.0–10.1)	7,700	9.4 (8.3–10.5)	9,300	0.8 (0.5–1.1)
≥75,000	13,600	8.0 (7.3–8.8)	12,600	8.2 (7.4–9.0)	15,800	0.9 (0.7–1.1)
**Employment status**						
Full-time	27,700	9.1 (8.6–9.7)	26,600	9.2 (8.7–9.8)	26,900	1.2 (1.0–1.4)
Part-time	11,700	11.0 (10.1–12.0)	10,300	11.9 (11.0–13.0)	10,600	1.3 (1.0–1.7)
Unemployed	5,700	14.1 (12.6–15.7)	5,200	14.8 (13.2–16.5)	4,300	2.8 (2.1–3.6)
Other^h^	9,100	11.1 (10.0–12.3)	8,100	11.9 (10.7–13.2)	11,600	1.4 (1.1–1.8)

Abbreviations: CI, confidence interval; AI/AN, American Indian or Alaskan Native; NH/PI, Native Hawaiian or other Pacific Islander.

a Adults who met at least 3 of 7 DSM-IV (Diagnostic and Statistical Manual of Mental Disorders, Fourth Edition) alcohol dependence criteria (ie, tolerance, withdrawal, impaired control, unsuccessful attempts to cut down or stop drinking, continued use despite problems, neglect of activities, time spent in alcohol-related activity) and consumed 1 or more drinks on 6 or more days in the past 12 months. Respondents whose information was unknown were excluded.

b Data set used for 2010 was revised in March 2012.

c Adults who reported drinking 15 or more drinks per week (men) or 8 or more drinks per week (women) in the past 30 days; reported drinking 5 or more drinks (men) or 4 or more drinks (women) during an occasion on at least 1 day in the past 30 days; reported any drinking (adults aged 18–20) in the past 30 days and not included in the category of binge drinking; or reported any alcohol consumption and pregnancy (women) in the past 30 days.

d Adults who reported consuming 5 or more drinks (men) or 4 or more drinks (women), per occasion, on at least 1 day in the past 30 days.

e Adult drinkers who did not report consuming 5 or more drinks (men) or 4 or more drinks (women), per occasion, on at least 1 day in the past 30 days.

f Sample sizes of the number of survey respondents (rounded to the nearest hundred).

g Suppressed due to low precision.

h Students, people keeping house or caring for children full time, retired people or people with disabilities, or other people not in the labor force.

Among binge drinkers, the prevalence of alcohol dependence was significantly higher among men (11.1%; 95% CI, 10.6%–11.7%) than among women (9.7%; 95% CI, 9.0%–10.3%) (Table 2). It was also highest among binge drinkers aged 18 to 24 (13.2%; 95% CI, 12.7%–13.8%); it then decreased significantly with increasing age. Binge drinkers who were American Indians or Alaskan Natives had a significantly higher prevalence of alcohol dependence than those in other racial/ethnic groups (28.1%; 95% CI, 21.4%–36.0%), as did binge drinkers with less than a high school education (14.2%; 95% CI, 12.9%–15.7%), an annual family income of less than $25,000 (14.9%; 95% CI, 13.9%–15.9%), and those who were unemployed (14.8%; 95% CI, 13.2%–16.5%), compared with those in other groups in the same sociodemographic category (ie, education, income, and employment).

The prevalence of alcohol dependence increased significantly with the frequency of binge drinking in the past month, ranging from 4.3% (95% CI, 3.9%–4.8%) among those who reported binge drinking 1 or 2 times in the past month to 29.8% (95% CI, 28.1%–31.9%) among those who reported binge drinking 10 or more times in the past month (Figure).

**Figure:**
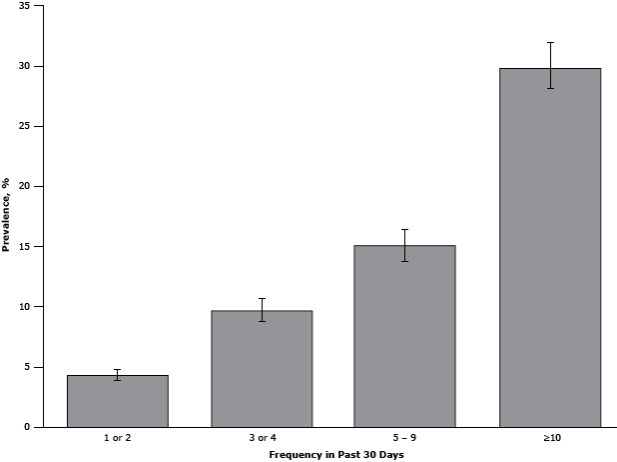
Prevalence of alcohol dependence among US adult binge drinkers, by binge drinking frequency during the past 30 days, National Survey on Drug Use and Health, 2009–2011. Alcohol dependence for adults was defined as meeting at least 3 of 7 criteria for alcohol dependence (ie, tolerance, withdrawal, impaired control, unsuccessful attempts to cut down or stop drinking, continued use despite problems, neglect of activities, time spent in alcohol-related activity) in the DSM-IV (Diagnostic and Statistical Manual of Mental Disorders, Fourth Edition) and consumed at least 1 drink on 6 or more days in the past 12 months. Binge drinking was defined as consuming 5 or more drinks (men) or 4 or more drinks (women) during a single occasion in the past 30 days. Data set used for 2010 was revised in March 2012. Error bars indicate 95% confidence intervals. **Table:** 

Binge Drinking Frequency in Past 30 Days	Prevalence, % (95% Confidence Interval)
1 or 2 times	4.3 (3.9–4.8)
3 or 4 times	9.7 (8.8–10.7)
5–9 times	15.1 (13.8–16.4)
≥10 times	29.8 (28.1–31.9)

## Discussion

This study found that about 9 of 10 adult excessive drinkers did not meet the diagnostic criteria for alcohol dependence. About 90% of the adults who drank excessively reported binge drinking, and the prevalence of alcohol dependence was similar among excessive drinkers and binge drinkers across most sociodemographic groups. The prevalence of alcohol dependence also increased with the frequency of binge drinking. However, even among those who reported binge drinking 10 or more times in the past month, more than two-thirds did not meet diagnostic criteria for alcohol dependence according to their responses to the survey.

The prevalence of alcohol dependence among adult excessive drinkers and binge drinkers in this study was slightly higher than the prevalence reported in other studies using the same diagnostic criteria for the classification of alcohol dependence. A 2001 study of alcohol abuse and dependence among US adults using the National Household Survey on Drug Abuse (NHSDA) — the precursor to the NSDUH — found that the prevalence of alcohol dependence was 7.4% among men and 7.3% among women who reported binge drinking (13). The higher prevalence of alcohol dependence among binge drinkers in this study may be due to the different time period as well as differences in the survey methods, which make these estimates not directly comparable (14). The former NHSDA was redesigned in 1999, and other changes were made to the survey in 2002, which may have increased the sensitivity of the NSDUH for identifying people who are binge drinking and alcohol dependent.

Differences in the prevalence of alcohol dependence between the current study and the 2002 study in New Mexico (10.7% among excessive drinkers and 8.1% among binge drinkers) (8) are probably due to differences in the survey methods used by the NSDUH and the BRFSS as well as differences in the populations studied (15).

Consistent with previous studies, binge drinking was most common among men, those aged 18 to 24, non-Hispanics whites, those with some college education, and those with an annual family income $75,000 or more (16). In contrast, alcohol dependence was most common among American Indians or Alaskan Natives, those having less than a high school education, and those with an annual family income of less than $25,000. These findings may reflect the known impact of alcohol dependence on many areas in the drinker’s life, including their ability to work and their productivity in the workplace. Reduced workplace productivity is the single largest contributor to alcohol-attributable economic costs in the United States (1).

The strong relationship between the prevalence of excessive drinking and binge drinking is also consistent with the findings of previous studies (17), as is the higher prevalence of alcohol dependence among binge drinkers relative to all current drinkers (8), and the positive relationship between the frequency of binge drinking and alcohol dependence (18). These findings emphasize the usefulness of screening for binge-level alcohol consumption to identify excessive drinking among adults, including those who are alcohol-dependent (19). The relatively low prevalence of alcohol dependence among people who drink excessively also suggests that most people who are screened for excessive drinking in clinical settings will probably not need to be referred for specialized treatment.

This study has several limitations. First, data for this study are based on self-reported alcohol consumption, which tends to be underreported (20). However, NSDUH estimates of the prevalence of binge drinking among adults are significantly higher than those from other surveys of adults, such as the BRFSS (16). In addition, although the classification of alcohol dependence in this study is based on self-reported responses to a survey, the questions used to assess substance use disorders in the NSDUH have been found to be sensitive and specific for identifying alcohol dependence among adults when tested in clinical settings (21). Second, although the NSDUH collects information from residents of households and noninstitutional group quarters (eg, shelters, rooming houses, dormitories) and from civilians living on military bases, the survey does not include homeless persons who were not living in shelters, military personnel on active duty, and residents of institutional group quarters (eg, jails, long-term care facilities), and the prevalence of excessive drinking — including alcohol dependence — is known to be higher in some of these populations (22,23). Third, the 2009–2011 NSDUH classified alcohol dependence by using DSM-IV criteria. In contrast, the new Diagnostic and Statistical Manual of Mental Disorders, Version 5 (DSM-5) defines a spectrum of alcohol use disorders, and based on these diagnostic criteria, alcohol dependence would be classified as a severe alcohol use disorder. However, a study of patients with a known substance use disorder found that the prevalence of severe alcohol use disorders was only 0.5% higher than the prevalence of alcohol dependence (24). Another study assessing the drinking behavior of the general population (using data from the 2004–2005 NESARC survey) estimated that the prevalence of severe alcohol use disorders based on DSM-5 diagnostic criteria was only about 1.1% higher than the prevalence of alcohol dependence according to DSM-IV criteria (25). Taken together, these findings suggest that the use of DSM-IV diagnostic criteria for alcohol dependence in this study probably resulted in only a slightly lower estimate of the prevalence of severe alcohol problems than would have been obtained using DSM-5 diagnostic criteria.

The findings of this study have important implications for planning and implementing public health interventions to reduce excessive drinking and binge drinking at the population level. Although alcohol dependence is an important public health problem, these findings suggest that most excessive drinkers are unlikely to need addiction treatment. Studies have also found that binge drinking, in particular, is strongly affected by alcohol policies in states (26). The Community Preventive Services Task Force recommends several policy strategies for reducing excessive alcohol use and related harms that are likely to be effective across many sociodemographic categories, including increasing alcohol taxes, regulating alcohol outlet density, and dram shop (commercial host) liability (27).

The US Preventive Services Task Force also recommends alcohol screening and brief counseling for excessive alcohol use among adults in primary care settings (28). However, recent studies have found that these prevention strategies are underused — probably because of timing constraints, lack of training, discomfort with discussing substance use, or insurance coverage limitations. The uptake of alcohol screening and brief interventions might be improved by offering health care providers more training opportunities and by including coverage for alcohol screening and brief interventions in standard health insurance plans (19,29). A comprehensive approach to reducing excessive alcohol use that emphasizes the implementation of effective policy strategies and clinical preventive services, similar to the *Best Practices for Comprehensive Tobacco Control Programs — 2007* (30), might therefore be expected to have a greater impact on reducing excessive alcohol use and related harms than a more focused strategy that primarily relies on the implementation of addiction treatment services alone.

## Figures and Tables

**Table:** 

**1. The activity supported the learning objectives.**				
**Strongly Disagree**				**Strongly Agree**
1	2	3	4	5
**2. The material was organized clearly for learning to occur.**				
**Strongly Disagree**				**Strongly Agree**
1	2	3	4	5
**3. The content learned from this activity will impact my practice.**				
**Strongly Disagree**				**Strongly Agree**
1	2	3	4	5
**4. The activity was presented objectively and free of commercial bias.**				
**Strongly Disagree**				**Strongly Agree**
1	2	3	4	5

## Post-Test Information

To obtain credit, you should first read the journal article. After reading the article, you should be able to answer the following, related, multiple-choice questions. To complete the questions (with a minimum 75% passing score) and earn continuing medical education (CME) credit, please go to http://www.medscape.org/journal/pcd. Credit cannot be obtained for tests completed on paper, although you may use the worksheet below to keep a record of your answers. You must be a registered user on Medscape.org. If you are not registered on Medscape.org, please click on the "Register" link on the right hand side of the website to register. Only one answer is correct for each question. Once you successfully answer all post-test questions you will be able to view and/or print your certificate. For questions regarding the content of this activity, contact the accredited provider, CME@medscape.net. For technical assistance, contact CME@webmd.net. American Medical Association's Physician's Recognition Award (AMA PRA) credits are accepted in the US as evidence of participation in CME activities. For further information on this award, please refer to http://www.ama-assn.org/ama/pub/about-ama/awards/ama-physicians-recognition-award.page. The AMA has determined that physicians not licensed in the US who participate in this CME activity are eligible for **AMA PRA Category 1 Credits**™. Through agreements that the AMA has made with agencies in some countries, AMA PRA credit may be acceptable as evidence of participation in CME activities. If you are not licensed in the US, please complete the questions online, print the AMA PRA CME credit certificate and present it to your national medical association for review.

## Post-Test Questions

### Article Title: Prevalence of Alcohol Dependence Among US Adult Drinkers, 2009–2011

**CME Questions**

1. You are consulting for a large health maintenance organization on prevention of excessive alcohol consumption. According to the database study of survey responses by Esser and colleagues, which of the following statements about excessive drinking and clinical correlates among adult drinkers in the United States is ***correct***?
  - Excessive drinking is most common among middle-aged women
  - Approximately half of excessive drinkers meet at least 3 of 7 *Diagnostic and Statistical Manual of Mental Disorders, Fourth Edition*, criteria for alcohol dependence
  - A comprehensive approach emphasizing evidence-based policy strategies and clinical preventive services could help to reduce excessive drinking
  - The prevalence of past-month excessive drinking was 15%
2. According to the database study of survey responses by Esser and colleagues, which of the following statements about binge drinking and clinical correlates among adult drinkers in the United States is ***correct***?
  - Binge drinking was most prevalent among men and among those 18 to 24 years old
  - Binge drinking was most prevalent among those with annual family incomes of less than $25,000
  - The prevalence of past-month binge drinking was 9%
  - Blacks had a significantly higher prevalence of binge drinking than did other racial and ethnic groups
3. According to the database study of survey responses by Esser and colleagues, which of the following statements about alcohol dependence and clinical correlates among adult drinkers in the United States would ***most*** likely be correct?
  - The prevalence of alcohol dependence was 10.2% among excessive drinkers, 10.5% among binge drinkers, and 1.3% among nonbinge drinkers
  - There was no association between alcohol dependence and binge-drinking frequency
  - Alcohol dependence was most prevalent among those with annual family incomes of $26,000 to $74,000
  - The prevalence of alcohol dependence among current drinkers was significantly higher among Native Hawaiians or other Pacific Islanders than among other racial/ethnic groups

**Evaluation**
